# Dissecção Coronária Espontânea em Paciente com Xantomatose Cerebrotendinosa

**DOI:** 10.36660/abc.20190456

**Published:** 2020-09-11

**Authors:** Maria Júlia Silveira Souto, Marcos Antônio Almeida-Santos, Eduardo José Pereira Ferreira, Luiz Flávio Galvão Gonçalves, Joselina Luzia Menezes Oliveira, Antônio Carlos Sobral Sousa

**Affiliations:** 1 Universidade Federal de Sergipe São CristovãoSE Brasil Universidade Federal de Sergipe, São Cristovão, SE - Brasil; 2 Programa de Pós-Graduação em Saúde e Meio Ambiente Universidade Tiradentes AracajuSE Brasil Programa de Pós-Graduação em Saúde e Meio Ambiente, Universidade Tiradentes, Aracaju, SE - Brasil; 3 Centro de Educação e Pesquisa Fundação São Lucas AracajuSE Brasil Centro de Educação e Pesquisa da Fundação São Lucas, Aracaju, SE – Brasil

**Keywords:** Xantomatose Cerebrotendinosa, Colesterol, Colestanol, Ácido Quenodesoxicólico/efeitos adversos, Doença da Artéria Coronariana/cirurgia, Diagnóstico por Imagem, Criança, Adolescente

## Introdução

A Xantomatose Cerebrotendinosa (XCT) é uma doença autossômica recessiva caracterizada pela formação de lesões xantomatosas em muitos tecidos, em particular no cérebro e tendões.^1^ O distúrbio é consequência da redução da produção de ácidos biliares, predominantemente do ácido quenodesoxicólico (CDCA) e do aumento da formação de colestanol.^2^ Manifestações clínicas comuns incluem diarreia infantil e catarata bilateral de início juvenil, geralmente seguida por xantomas tendinosos e disfunção neurológica progressiva.^3^ O diagnóstico final é baseado em anormalidades bioquímicas, incluindo níveis plasmáticos elevados de colestanol e aumento dos níveis urinários de álcool biliar associados a uma concentração biliar diminuída de CDCA.^4^ O tratamento é baseado na suplementação oral de CDCA que, se iniciada precocemente, pode prevenir grandes problemas clínicos, uma vez que produz uma redução na síntese e nos níveis plasmáticos de colestanol.^3^

O comprometimento cardiovascular em pacientes com XCT é principalmente associado à aterosclerose prematura.^4^ A análise lipídica no sangue de pacientes com XCT revelou níveis dramaticamente altos de 27-hidroxicolesterol e baixos níveis de lipoproteína de alta densidade-colesterol (HDL), que colocam esses pacientes em alto risco de doença cardiovascular.^5^

A dissecção espontânea da artéria coronária (SCAD, do inglês *spontaneous coronary artery dissection)* é definida como uma separação não traumática da parefde arterial coronariana, criando um falso lúmen, o que leva a uma redução do fluxo sanguíneo.^6^ Embora existam outras condições sistêmicas que tornam a parede do vaso coronariano vulnerável a essa condição, em pacientes com doença arterial coronariana aterosclerótica, a ruptura de um fibroateroma de capa fina pode levar à SCAD.^7^

Descrevemos um relato de caso de uma paciente com diagnóstico de XCT que apresentou comprometimento cardíaco devido à SCAD.

## Relato do caso

Em 2013, uma paciente do sexo feminino, de 22 anos, relatou história de xantomas no tendão de Aquiles e crise epiléptica parcial complexa nos últimos 10 anos. Ela evoluiu com dificuldade progressiva na capacidade de aprendizado e andar. Associada a essa apresentação clínica, ela relatou história de cirurgia bilateral para catarata aos 14 anos e esteatorreia.

**Figura 1:**
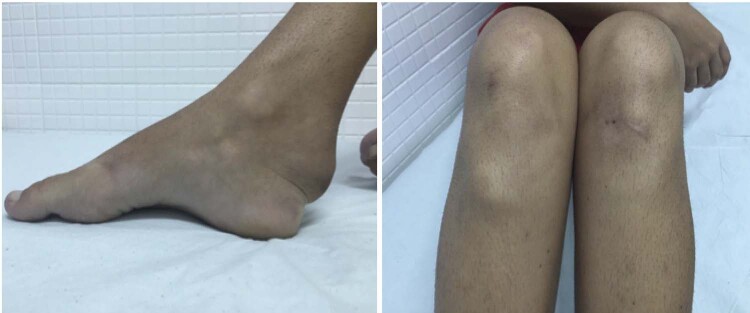
Xantomas observados na região do tendão de Aquiles direito e no joelho direito.

No exame físico, os xantomas foram observados principalmente na região do tendão de Aquiles, bilateralmente, mas também no cotovelo e joelho direitos ( Figura 1 ). O exame neurológico revelou discreta dismetria e disdiadococinesia, dificuldade na realização do teste de caminhada em linha reta e hiperreflexia patelar bilateral e simétrica. Não havia anormalidades nos exames de força ou sensibilidade.

A ressonância magnética do cérebro mostrou uma área focal de 1,4 cm, com hipersinal nas sequências ponderadas em T2 e hipossinal nas sequências ponderadas em T1, sem realce de contraste. O ecodopplercardiograma transtorácico mostrou dilatação moderada e disfunção ventricular esquerda regional, resultando em comprometimento moderado da função sistólica e insuficiência mitral leve. A ultrassonografia abdominal demonstrou a presença de colelitíase.

A paciente, portanto, apresentava achados clínicos e radiológicos compatíveis com a XCT. O diagnóstico foi confirmado pela descoberta de um nível sérico elevado de colestanol de 31,79 mcg/mL. Ela iniciou o tratamento com CDCA no mesmo ano.

**Figura 2:**
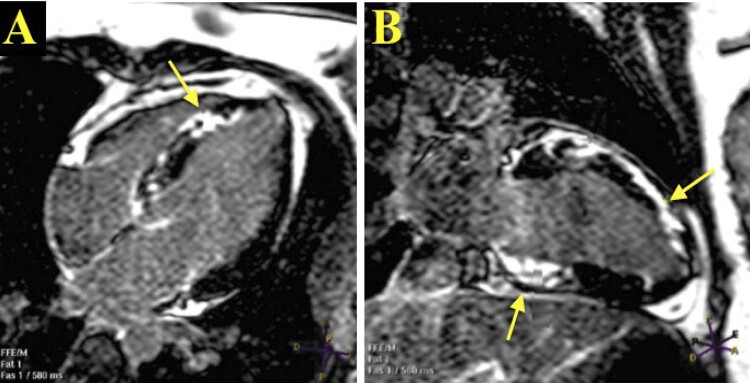
– Ressonância magnética apresentando realce tardio transmural com gadolínio (setas) das paredes médio-basal inferior, anterior e septo-anterior do ventrículo esquerdo em projeção de quatro (A) e duas câmaras (B).

Em 2017, ela foi submetida a um novo exame cardiovascular. Uma ressonância magnética cardíaca foi realizada e revelou um ventrículo esquerdo dilatado, associado a disfunção ventricular esquerda leve (fração de ejeção do ventrículo esquerdo = 47%) como consequência de acinesia da parede basal média inferior e discinesia nas paredes anterior e anterior-septal do ventrículo esquerdo. Essas regiões apresentaram comprometimento da perfusão na avaliação dinâmica baseada em gadolínio e presença de realce tardio transmural com gadolínio ( Figura 2 ).

**Figura 3:**
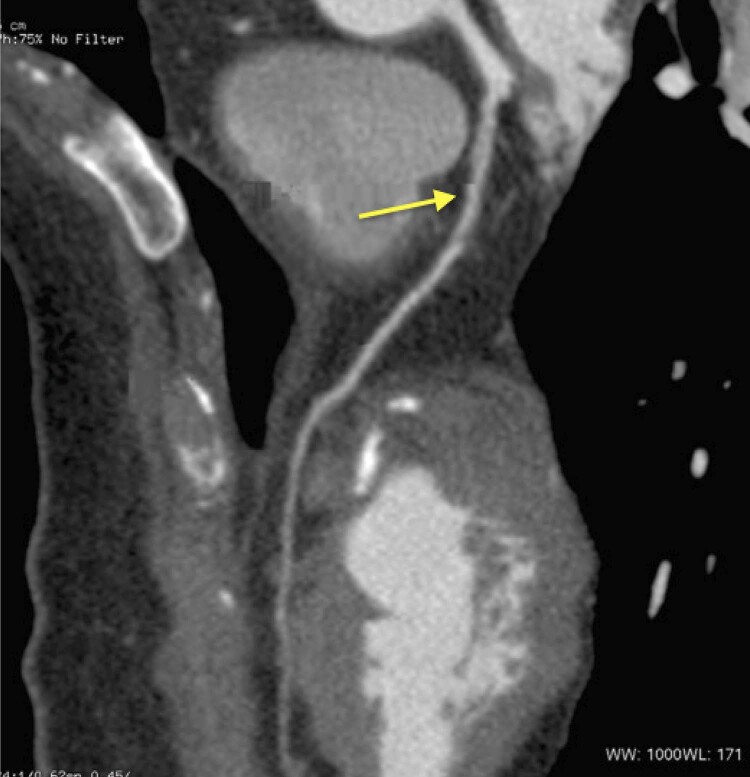
– Reconstrução multiplanar da angiotomografia computadorizada das coronárias, detectando uma irregularidade parietal grave no terço proximal da artéria coronária descendente anterior, o que sugeria a presença de uma placa não calcificada ou dissecção da artéria (seta).

A angiotomografia coronária detectou uma irregularidade parietal grave no terço proximal da artéria descendente anterior (ADA) com redução luminal de 50%, o que sugeria a presença de placa não calcificada ou dissecção da artéria ( Figura 3 ).

**Figura 4:**
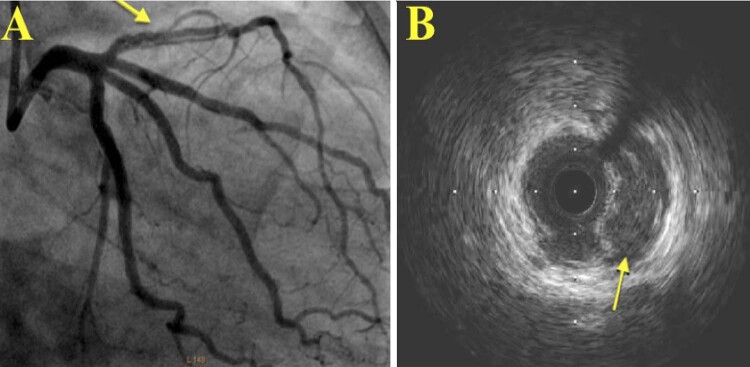
– A - Angiografia coronária da artéria coronária esquerda apresentando dissecção no terço proximal e médio da artéria descendente anterior esquerda (seta). B - Ultrassonografia intracoronária com sinal de duplo lúmen na artéria descendente anterior esquerda (seta).

Esta última foi confirmada por angiografia coronária e ultrassonografia intracoronária, que mostraram dissecção nos terços medial e proximal da ADA sem comprometimento do fluxo distal ( Figura 4 ).

No momento do diagnóstico, seu painel lipídico era: colesterol total 170 mg/dL; lipoproteína de alta densidade-colesterol (HDL-C) 47 mg/dL; lipoproteína de baixa densidade-colesterol (LDL-C) 101 mg/dL; triglicérides 108 mg/dL.

Com base nesses achados, a paciente iniciou terapia cardiovascular com Ramipril 10 mg por dia, aspirina 100 mg por dia, carvedilol 6,25 mg duas vezes ao dia e rosuvastatina 10 mg na hora de dormir, associados à manutenção da suplementação oral de ácido biliar com CDCA.

## Discussão e Conclusões

A Xantomatose Cerebrotendinosa é causada por uma mutação em homozigose da enzima mitocondrial esterol 27-hidroxilase (CYP27), a qual leva a várias manifestações sistêmicas.^8^ O diagnóstico é estabelecido com o reconhecimento desses sintomas e pelo achado de colestanol plasmático elevado e, se possível, um diagnóstico definitivo é obtido pela análise molecular do gene CYP27A1.^9 , 10^ No presente caso, o diagnóstico de XCT foi estabelecido com base na forte sintomatologia associada aos níveis plasmáticos de colestanol, muito semelhantes à concentração sérica média em outros estudos (31,79 mcg/mL).^4 , 10^

As manifestações cardíacas são menos notáveis e se apresentam principalmente como doença coronariana grave, incluindo infarto do miocárdio, angina pectoris, doença arterial coronariana e alterações isquêmicas no eletrocardiograma.^5 , 11^ Posteriormente, dois grandes estudos realizados por Duell et al.,^10^ e Sekijima et al.,^12^ demonstraram a presença de doença cardiovascular associada à XCT apenas em 7% e 20% de seus pacientes, respectivamente. No caso relatado, estudamos uma paciente com XCT que desenvolveu doença arterial coronariana causada por SCAD. Embora várias situações clínicas específicas, incluindo displasia fibromuscular e gravidez, tenham sido principalmente associadas à SCAD, as condições ateroscleróticas também podem estar relacionadas à patogênese dessa doença.^6^ Uma vez que a XCT predispõe ao desenvolvimento de aterosclerose prematura e existem poucos estudos que relatam doença arterial coronariana associada a tromboembolismo aterosclerótico,^5^ há evidências de que a SCAD no caso relatado também estava associada a uma placa ateromatosa. Até onde os autores puderam investigar, este é provavelmente o primeiro caso na literatura que demonstra a associação entre XCT e SCAD.
