# Supplementary material for: Dissecção Coronária Espontânea em Paciente com Xantomatose Cerebrotendinosa
Source: Arq Bras Cardiol. 2020 Sep 11;115(1 Suppl 1):18–21. [Article in Portuguese] doi: 10.36660/abc.20190456 (PMC8386949; doi:10.36660/abc.20190456)
Supplement: Translation

# Translation: Spontaneous Coronary Artery Dissection in a Patient with Cerebrotendinous Xanthomatosis

**Authors:** Maria Júlia Silveira Souto, Marcos Antônio Almeida-Santos, Eduardo José Pereira Ferreira, Luiz Flávio Galvão Gonçalves, Joselina Luzia Menezes Oliveira, Antônio Carlos Sobral Sousa

**Affiliations:** 1 Brazil Universidade Federal de Sergipe, São Cristovão, SE - Brazil; 2 Brazil Programa de Pós-Graduação em Saúde e Meio Ambiente, Universidade Tiradentes, Aracaju, SE - Brazil; 3 Brazil Centro de Educação e Pesquisa da Fundação São Lucas, Aracaju, SE – Brazil

**Keywords:** Xanthomatosis Cerebrotendinous, Cholesterol, Cholestanol, Chenodeoxycholic Acid/adverse effects, Diagnosis, Imaging, Child, Adolescent

## Introduction

Cerebrotendinous xanthomatosis (CTX) is an autosomal recessive disease characterized by the formation of xanthomatous lesions in many tissues, particularly the brain and tendons.^1^ The disorder is a consequence of the reduced production of bile acids, predominantly chenodeoxycholic acid (CDCA), and an increased formation of cholestanol.^2^ Common clinical manifestations include infant-onset diarrhea and juvenile-onset bilateral cataract, usually followed by tendon xanthomas and progressive neurological dysfunction.^3^ The final diagnosis is based on biochemical abnormalities, including elevated plasma cholestanol level and increased levels of bile alcohol in urine associated with a diminished biliary concentration of CDCA.^4^ The treatment is based on oral supplementation of CDCA, which, if initiated early, can prevent major clinical problems, as it produces a reduction in cholestanol synthesis and plasma levels.^3^

Cardiovascular impairment in patients with CTX is mostly associated with premature atherosclerosis.^4^ Blood lipid analysis in patients with CTX revealed dramatically high levels of 27-hydroxycholesterol and low levels of high-density lipoprotein cholesterol (HDL), which place these patients at a high risk of cardiovascular disease.^5^

Spontaneous coronary artery dissection (SCAD) is defined as a non-traumatic separation of the coronary arterial wall, creating a false lumen, which leads to blood flow reduction.^6^ Although there are other systemic conditions that make the coronary vessel wall vulnerable to this condition, in patients with atherosclerotic coronary artery disease, the rupture of a thin-cap fibroatheroma might lead to SCAD.^7^

We describe a case report of a patient diagnosed with CTX who showed cardiac impairment due to SCAD.

## Case report

In 2013, a female patient, 22 years old, reported a history of xanthomas in the Achilles tendon and complex partial epileptic crisis for the last 10 years. She developed progressive difficulty in learning and walking skills. Associated to this clinical presentation, she reported a history of bilateral surgery for cataract when she was 14 years old and steatorrhea.

On physical examination, xanthomas were observed mostly on the region of the Achilles tendon, bilaterally, but also on the right elbow and knee ( Figure 1 ). The neurological exam showed mild dysmetria and dysdiadochokinesia, difficulty performing the straight line walking test, and bilateral and symmetric patellar hyperreflexia. There was no abnormality on strength or sensitivity exams.

**Figure 1:** – Xanthomas observed on the region of the right Achilles tendon and on the right knee.

The magnetic resonance imaging of the brain showed a focal area of 1.4 cm, with hypersignal in T2-weighted and hyposignal in T1-weighted sequences, with no contrast enhancement. The transthoracic echodopplercardiogram found a moderate left ventricular dilatation and regional dysfunction, resulting in a moderate impairment in its systolic function, and a mild mitral regurgitation. The abdominal ultrasound showed cholelithiasis.

The patient, therefore, had clinical and radiological findings compatible with CTX. The diagnosis was confirmed by an elevated serum cholestanol level of 31.79 mcg/mL. She started the treatment with CDCA in the same year.

In 2017, she was submitted to a new cardiovascular examination. A cardiac magnetic resonance imaging was performed and showed a dilated left ventricle, associated with mild left ventricular dysfunction (left ventricular ejection fraction = 47%), as a consequence of akinesia of the inferior medium-basal wall and dyskinesia in the anterior and anterior-septal walls of the left ventricle. These regions showed perfusion impairment in the gadolinium-based dynamic evaluation and the presence of transmural late gadolinium enhancement ( Figure 2 ).

**Figure 2:** – Magnetic resonance imaging showing transmural late gadolinium enhancement (arrows) of the inferior medium-basal, anterior and anterior-septal walls of the left ventricle in four-chamber (A) and two-chamber views (B).

The coronary computed tomographic angiography detected severe parietal irregularity in the proximal third of the anterior descending coronary artery (LAD) with luminal reduction of 50%, which suggested the presence of a noncalcified plaque or dissection of the artery ( Figure 3 ).

**Figure 3:** – Multiplanar reformation of coronary computed tomographic angiography, detecting a severe parietal irregularity in the proximal third of the anterior descending coronary artery, which suggested the presence of a noncalcified plaque or dissection of the artery (arrow).

The latter was confirmed by coronary angiography and intracoronary ultrasound, which showed a dissection in the medial and proximal thirds of the LAD, with no impairment of the distal flow ( Figure 4 ).

**Figure 4:** – A - Coronary angiography of left coronary showing dissection in the proximal and medium third of left anterior descending artery (arrow). B- Intracoronary ultrasound showing double-lumen sign in the left anterior descending artery (arrow).

At the time of diagnosis, her lipid panel was: total cholesterol 170 mg/dL; high-density lipoprotein cholesterol (HDL-C) 47 mg/dL; low-density lipoprotein cholesterol (LDL-C) 101 mg/dL; triglycerides 108 mg/dL.

Based on these findings, the patient was started on cardiovascular therapy with Ramipril 10 mg per day, Aspirin 100 mg per day, Carvedilol 6.25 mg twice a day and Rosuvastatin 10 mg at bedtime, associated to the maintenance of the oral bile acid supplementation with CDCA.

## Discussion and Conclusions

Cerebrotendinous xanthomatosis is caused by a homozygous mutation of the mitochondrial enzyme sterol 27-hydroxylase (CYP27), which leads to a number of systemic manifestations.^8^ The diagnosis is established upon recognition of these symptoms and the finding of elevated plasma cholestanol, and, if possible, a definitive diagnosis is obtained through the molecular analysis of CYP27A1 gene.^9 , 10^ In the present case, the diagnosis of CTX was established based on the strong symptomatology associated with plasma cholestanol levels, which were very similar to the mean serum concentrations found in other studies (31.79 mcg/mL).^4 , 10^

Cardiac manifestations are less remarkable and present mostly as severe coronary disease, including myocardial infarction, angina pectoris, coronary artery disease and ischemic changes on the electrocardiogram.^5 , 11^ Subsequently, two large studies carried out by Duell et al.^10^ and Sekijima et al.^12^ demonstrated the presence of cardiovascular disease associated to CTX only in 7% and 20% of their patients, respectively. In this case report, we studied a patient with CTX who developed coronary artery disease due to SCAD. Although several specific clinical situations, including fibromuscular dysplasia and pregnancy, have been mostly associated with SCAD, atherosclerotic conditions may be as well related to the pathogenesis of this disease.^6^ As the CTX predisposes to the development of premature atherosclerosis and there are a few studies that report coronary artery disease associated to atherosclerotic thromboembolism,^5^ there is evidence that the SCAD in the reported case was also associated to an atheromatous plaque formation. As far as the authors could investigate, this is probably the first case in the literature demonstrating the association between CTX and SCAD.
